# Low Intraoperative Cerebral Oxygen Saturation Is Associated with Acute Kidney Injury after Off-Pump Coronary Artery Bypass

**DOI:** 10.3390/jcm12010359

**Published:** 2023-01-02

**Authors:** Seo Hee Ko, Jong-Wook Song, Jae-Kwang Shim, Sarah Soh, Young-Lan Kwak

**Affiliations:** 1 Department of Anesthesiology and Pain Medicine, Yonsei University College of Medicine, 50-1 Yonsei-ro, Seodaemun-Gu, Seoul 03722, Republic of Korea; 2 Anesthesia and Pain Research Institute, Yonsei University College of Medicine, 50-1 Yonsei-ro, Seodaemun-Gu, Seoul 03722, Republic of Korea

**Keywords:** regional cerebral oxygen saturation, cerebral oximetry, near-infrared spectroscopy, acute kidney injury, off-pump coronary artery bypass

## Abstract

By monitoring the brain as the index organ of global oxygen supply–demand balance including major organs, regional cerebral oxygen saturation (rScO_2_) may indicate adequacy of renal perfusion. The aim of this study was to investigate the relationship between perioperative rScO_2_ and acute kidney injury (AKI) after off-pump coronary artery bypass (OPCAB). AKI was diagnosed according to the Kidney Disease: Improving Global Outcomes criteria. Collected rScO_2_ variables were baseline, mean, and lowest value during surgery, maximal percentage decrease from baseline, and areas under the threshold below an absolute value of 50% (AUT_50_) and of 80% of baseline (AUT_80%base_). Among 580 patients, AKI developed in 143 (24.7%) patients. Patients with AKI had lower baseline, mean, and lowest rScO_2_ and higher AUT_50_ and AUT_80%base_ than those without AKI despite routine efforts to restore the rScO_2_ values within 20% of the baseline. Among the rScO_2_ variables, the area under the receiver operating characteristic curve of mean rScO_2_ was the highest (0.636), which was used for the multivariable logistic regression. Multivariable logistic regression revealed mean rScO_2_ as an independent predictor of AKI (odds ratio, 0.964; 95% confidence interval, 0.937–0.990; *p* = 0.008), along with chronic kidney disease and emergency surgery. Low intraoperative mean rScO_2_ was independently associated with AKI after OPCAB, which may serve as an early marker of renal injury.

## 1. Introduction

Acute kidney injury (AKI) is a prevalent complication of cardiac surgery leading to increased risks of chronic kidney disease (CKD) and mortality [1]. Due to lack of definitive therapeutic strategies, early detection and preventive measures have been primary goals of AKI management [2,3]. None of the available biomarkers of AKI serve this purpose, although renal near-infrared spectroscopy (NIRS) has been suggested as a continuous real-time monitoring technique to detect instant changes in renal biomarkers related to the adequacy of renal perfusion [4,5]. Relevant clinical studies in cardiac surgeries, however, have shown conflicting results [6,7]. This is probably in association with a fundamental technical limitation regarding the monitoring depth of NIRS (2.0–2.5 cm) [8], which is shallower than the skin-to-kidney depth in most patients [6].

Without critical restrictions on application, regional cerebral oxygen saturation (rScO_2_) has demonstrated significant correlations with left ventricular ejection fraction (LVEF) and mixed venous oxygen saturation (SvO_2_), indicating that rScO_2_ could reflect systemic oxygen supply–demand balance beyond that of the forebrain [9,10,11]. As the renal medulla is most vulnerable to hypoxia, a low rScO_2_ value would also serve as an early warning sign of inadequate renal perfusion and injury. In addition, baseline rScO_2_ was an important prognostic factor in adult cardiac surgery to indicate the circulatory reserve of the patient [10].

Studies have explored the association between rScO_2_ and AKI in cardiac surgical patients, but the results were inconclusive [7,12,13]. These studies were mostly underpowered and addressed surgeries using cardiopulmonary bypass (CPB). Differences in pH and hemodilution management during CPB would exert significant changes in hemoglobin (Hb) and arterial partial pressure of CO_2_, two main determinants of rScO_2_, and may influence rScO_2_ regardless of the systemic oxygenation balance [14,15,16]. This could complicate identification of the correlation between rScO_2_ and AKI in cardiac surgeries using CPB.

As off-pump coronary artery bypass surgery (OPCAB) avoids CPB, this correlation should be more obvious, although AKI remains a threat in association with hemodynamic instability during grafting [17,18]. Moreover, validation of an association between rScO_2_ and AKI might allow early prediction of AKI and early intervention in high-risk patients. Thus, we aimed to evaluate the relationship between perioperative rScO_2_ and AKI in OPCAB in this retrospective single-center cohort study.

## 2. Materials and Methods

### 2.1. Study Population

The electronic medical records of patients who underwent OPCAB between November 2016 and December 2020 at Severance Cardiovascular Hospital of Yonsei University Health System, Seoul, South Korea were retrospectively reviewed. OPCAB is the default strategy for surgical, isolated coronary revascularization at our institution. The study protocol followed the principles of Declaration of Helsinki and was approved by the Institutional Review Board of the Yonsei University Health System (4-2022-0322). The requirement for obtaining informed consent from the patients was waived. This manuscript adheres to the applicable STROBE guidelines for observational studies [19]. Patients who underwent minimally invasive surgery via thoracotomy, conversion to an on-pump procedure, or a combination of surgeries other than coronary revascularization and patients with incomplete data were excluded. Data from patients with rScO_2_ values not recorded for more than 10 min were considered incomplete. Patients with pre-existing AKI according to the Kidney Disease: Improving Global Outcomes (KDIGO) criteria [20] or undergoing preoperative renal replacement therapy also were excluded.

### 2.2. NIRS Measurement

The rScO_2_ was monitored with NIRS (INVOS Cerebral/Somatic Oximeter 5100; Covidien, Dublin, Ireland). The NIRS sensors were placed on both sides of the forehead (>3 cm above the eyebrows) before induction of anesthesia. The rScO_2_ was measured every 30 s during the anesthetic period. The baseline rScO_2_ was measured in the supine position without supplementary oxygen before anesthetics were administered. Collected rScO_2_ data were analyzed using INVOS Analytics Tools software (Covidien, Boulder, USA). The rScO_2_ variables included in the analysis were baseline value, mean value, lowest value during surgery, maximal percentage decrease from baseline, and area under the threshold (AUT) below an absolute value of 50% (AUT_50_) or below 80% of baseline (AUT_80%Base_). For analysis, left and right rScO_2_ values were obtained simultaneously, and the lower value was selected for analyzing baseline, mean, and lowest rScO_2_. The maximal percentage decrease from baseline rScO_2_ was that of the side with the greater reduction.

### 2.3. Clinical Data Assessment

Demographic data and preoperative clinical variables were collected from electrical medical records. Variables included diagnosed comorbidities such as hypertension, diabetes mellitus, CKD, myocardial infarction, unstable angina, congestive heart failure (New York Heart Association classification 3 or 4), left ventricular dysfunction (LVEF < 40%), cerebrovascular accident, and peripheral artery occlusive disease. Data on EuroSCORE II, preoperative laboratory values, medication, and emergency surgery were collected.

Assessed intraoperative data were duration of surgery; fluid balance; need for packed erythrocyte (pRBC) transfusion; urine output; reinfused cell salvage volume (surrogate of intraoperative blood loss); use of norepinephrine, vasopressin, or inotropes (milrinone and dobutamine); and cardiac index less than 2.0 L/min/m^2^ and SvO_2_ less than 65% during surgery.

### 2.4. Perioperative Management

All patients were managed by institutional standardized anesthetic and surgical protocols. Routine monitoring included pulmonary artery catheter, bispectral index score (BIS; A-200 BIS monitor; Aspect Medical System Inc., Norwood, MA, USA), transesophageal echocardiography, and rScO_2_. Anesthesia was induced with midazolam and sufentanil and sustained with sevoflurane and sufentanil infusion (BIS 40–60). Mean arterial pressure was targeted to at least 65–70 mmHg using norepinephrine (up to 0.3 μg/kg/min) first and adding vasopressin (up to 4 IU/h), if necessary. Milrinone (0.3–0.5 μg/min/kg) was administered if SvO_2_ was below 60% or cardiac index was below 2.0 L/min/m^2^ for more than 10 min. Allogeneic pRBC transfusion was considered at Hb concentrations lower than 7–8 g/dL or at the discretion of the attending anesthesiologist and cardiac surgeon.

When rScO_2_ showed a decrease of 20% or more from baseline, an institutional protocol using a previously proposed algorithm [21] was employed for intraoperative use of cerebral NIRS. First, check the sensor attachment, verify the head and neck position, and correct hypotension to increase cerebral perfusion pressure. If systemic oxygen saturation is low, supply a larger fraction of oxygen, increase end-tidal CO_2_ to the upper normal range, and consider pRBC transfusion if Hb concentration is less than 7–8 g/dL.

### 2.5. Endpoints

The primary endpoint of this study was to identify whether intraoperative rScO_2_ is independently associated with postoperative AKI. Postoperative AKI was defined as an increase in serum creatinine level ≥ 0.3 mg/dL from baseline (measured within 24 h before surgery (except in 9 patients: 48 h before surgery)) within 48 h, a 50% increase from baseline within 7 days, or urine volume less than 0.5 mL/kg/hour for more than 6 h [20].

Postoperative outcomes, including cerebrovascular accident, delirium, re-operation, sternal infection, prolonged mechanical ventilation for more than 24 h, myocardial infarction, the lengths of ICU and hospital stays, and 30-day or in-hospital mortality were recorded.

### 2.6. Statistical Analysis

All statistical analyses were performed using SPSS (version 25.0, IBM Corp, Armonk, NY, USA) and the R package, version 3.6.0 (The R Foundation for Statistical Computing, Vienna, Austria). Data are presented as mean (standard deviation, SD), median (interquartile range, IQR), or number (percentage). Normal distribution was assessed with the Shapiro–Wilk test. For intergroup comparisons, independent t-test, chi-square test, Fisher’s exact test, or Mann–Whitney U test were used as appropriate. Among various rScO_2_ parameters (baseline, mean, lowest rScO_2_, maximal percentage decrease, AUT_50_, and AUT_80%base_), the variable showing the highest predictive power for AKI development was determined by the area of receiver operating characteristic (AUROC) curve. Multivariable logistic regression analysis was used to identify the independent risk factors of AKI after OPCAB was constructed with mean rScO_2_ as it showed the highest predictive power of AKI among the rScO_2_ variables. The known risk factors and variables with a *p*-value < 0.01 from univariable analysis also were included in the multivariable model. To evaluate prediction performance of the multivariable model, the AUROC was calculated and the Hosmer–Lemeshow test was performed. The odds ratio (OR) and 95% confidence interval (CI) were calculated. Among these variables, there was no multicollinearity. The optimal cutoff values for mean rScO_2_ determined by AUROC analysis and Youden’s index and for perioperative parameters and postoperative outcomes were compared between the high and low mean rScO_2_ groups (divided by the cutoff value). In all analyses, *p* < 0.05 was considered statistically significant.

## 3. Results

**Figure 1:**
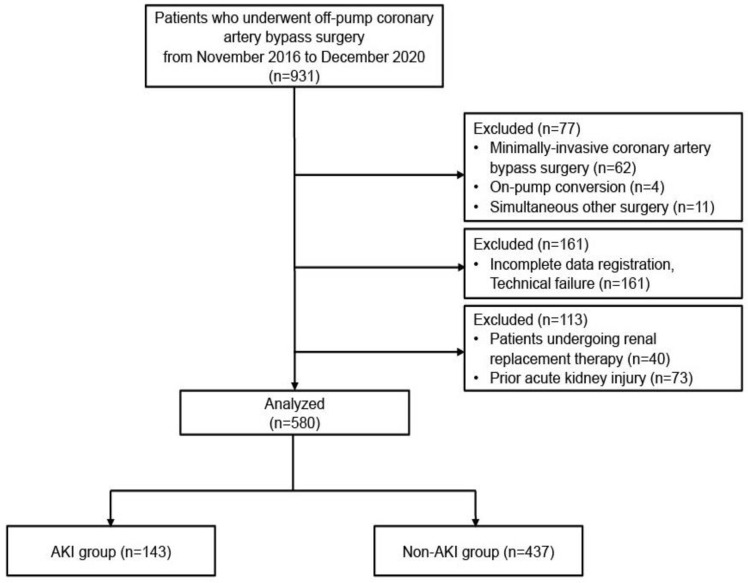
Flow diagram of the study.

Between November 2016 and December 2020, 931 patients underwent OPCAB. Of these, 580 patients fulfilled the study criteria for analysis (Figure 1).

Postoperative AKI occurred in 143 patients (24.7%). Patients in the AKI group were significantly older (*p* = 0.004) with a higher EuroSCORE II (*p* < 0.001) and preoperative CKD (*p* < 0.001), myocardial infarction within 1 week (*p* = 0.003), LVEF < 40% (*p* = 0.020), cerebrovascular accident (*p* = 0.035), and peripheral artery occlusive disease (*p* = 0.032) were more common compared to the non-AKI group. Anemia (*p* < 0.001) and emergency surgery (*p* < 0.001) were more frequent and albumin level (*p* < 0.001) was lower in the AKI group. In this group, intraoperative use of inotropes (*p* = 0.018), vasopressin (*p* = 0.017), and pRBC transfusion (*p* < 0.001) and the prevalence of low SvO_2_ during distal anastomosis (*p* = 0.040) were more frequent than in the non-AKI group (Table 1).

Longer ICU (*p* < 0.001) and hospital stays (*p* < 0.001) and a higher incidence of postoperative complications including delirium (*p* < 0.001), prolonged mechanical ventilation for longer than 24 h (*p* = 0.028), and 30-day or in-hospital mortality (*p* < 0.001) were observed in the AKI group compared to the non-AKI group (Supplementary Table S1).

The baseline, mean, and lowest rScO_2_ values during surgery were significantly lower and AUT_50_ was significantly higher in the AKI group than in the non-AKI group (all *p* < 0.001), whereas AUT_80%base_ was similar between the groups. The number of patients whose rScO_2_ values decreased below an absolute value of 50% was significantly greater in the AKI group than in the non-AKI group (*p* = 0.003) (Table 2).

Among the rScO_2_ variables, baseline, mean, and lowest rScO_2_ and AUT_50_ showed significant associations with AKI, and AUROC of mean rScO_2_ (0.636; 95% CI, 0.584–0.689) was highest, with a cutoff value of 58.5%, and was used in subsequent analysis.

In multivariable logistic regression analysis (no missing data among the assessed variables), the mean rScO_2_ value was an independent risk factor of AKI after OPCAB (OR, 0.964; 95% CI, 0.937–0.990; *p* = 0.008), along with CKD and emergency surgery (Table 3, Supplementary Table S2). The Hosmer–Lemeshow test indicated a good fit of the models (*p* = 0.940) and the apparent AUC of the model was 0.718 (95% CI, 0.669–0.767).

When patients were divided into groups based on a mean rScO_2_ of 58.5%, 301 (51.9%) were in the low mean rScO_2_ group. In this group, older age, larger number of females, higher EuroSCORE II, lower albumin level, and greater incidence of anemia, diabetes mellitus, CKD, CHF (all *p* < 0.001), and LVEF < 40% (*p* = 0.017) were observed compared to those in the high mean rScO_2_ group. Intraoperative use of vasopressin (*p* = 0.010) and pRBC transfusion (*p* < 0.001) were greater in the low mean rScO_2_ group than in the high mean rScO_2_ group. The prevalence of cardiac index < 2.0 L/min/m^2^ (*p* = 0.016) and SvO_2_ < 65% (*p* < 0.001) was significantly higher in the low mean rScO_2_ group than in the high mean rScO_2_ group (Supplementary Table S3).

Low mean rScO_2_ group showed significant association with the development of AKI (OR, 2.510; 95% CI, 1.683–3.745; *p* < 0.001). In addition, the incidence of cerebrovascular accident (*p* = 0.001) and delirium (*p* < 0.001) were higher, and the durations of ICU (*p* = 0.007) and hospital stay after surgery (*p* < 0.001) were longer in the low rScO_2_ group than in the high mean rScO_2_ group (Table 4).

## 4. Discussion

In this single-center retrospective study conducted in OPCAB patients, low mean rScO_2_ values demonstrated the best ability to predict postoperative AKI among various rScO_2_ parameters and were independently associated with postoperative AKI along with preoperative CKD and emergency surgery.

Early adaptation of postoperative KDIGO bundles in high-risk patients decreased the incidence of AKI and improved outcomes [3]. Thus, the importance of reliable early AKI biomarkers has been emphasized, although the most widely used serum creatinine biomarker lacks reliability. In contrast, rScO_2_ provides real-time information regarding regional tissue oxygenation, and its ability to detect adverse perioperative clinical outcomes beyond that of the cerebral outcome, using the brain as the index organ, has been evaluated [22,23]. From these, low rScO_2_ could reflect inadequate renal perfusion and oxygenation and be used as a marker of renal injury because renal ischemia is a critical pathophysiologic mechanism of AKI after cardiac surgery.

Of interest and relative to intraoperative rScO_2_ monitoring and management, OPCAB is a unique subset of cardiac surgery that avoids CPB, which is associated with discordant changes in rScO_2_ values due to hemodilution and temperature changes that may be independent of systemic perfusion and oxygenation. Moreover, in OPCAB, periods of mechanical cardiac constraint are accompanied by systemic hemodynamic deterioration that would be reflected by a decrease in rScO_2_, whether or not it is correctable by proposed strategies. Therefore, rScO_2_ values may reflect possible renal insult and would serve as a more reliable early biomarker of AKI after OPCAB than in surgeries requiring CPB. As AKI is still the most common complication after OPCAB and early identification of patients at risk would allow timely initiation of preventive measures such as the KDIGO bundle after surgery, finding such a relationship would be of influential clinical significance in the absence of other evidence.

In the present study, among various rScO_2_ values examined, intraoperative mean rScO_2_ was independently associated with AKI after OPCAB, exhibiting the greatest predictive power among the rScO_2_ variables. The baseline rScO_2_, lowest rScO_2_ and AUT_50_, but not AUT_80%base_, showed significant association with AKI but lower AUROCs. Similarly, patients experiencing major organ morbidity or mortality had lower baseline and mean rScO_2_ values [23], and low mean rScO_2_ was seen in patients developing any degree of renal dysfunction after cardiac surgery using CPB [13]. Likewise, the prognostic importance of baseline rScO_2_ in cardiac surgical patients was recommended to be used for risk stratification, while evidence was deemed insufficient to recommend the use of intraoperative rScO_2_ to reduce organ-specific morbidity [24,25]. Although AUT_50_ and AUT_80%base_ have been commonly used as definitions for desaturation in numerous studies performed in cardiac surgery using CPB, intraoperative decrease in rScO_2_, whether absolute or relative, was criticized for the following: an absolute cutoff value of rScO_2_ does not reference the baseline value, and the % decrease from baseline shows different ranges depending on the baseline value [7,10]. Nonetheless, the clinical value of intraoperative rScO_2_ monitoring was shown in terms of major organ dysfunction and mortality in a recent large cohort study [26]. In this context, the strength of the mean rScO_2_ value as the most prominent NIRS value of the current study is its reflection of both baseline and degree of intraoperative decline (regardless of relative decrease) as a single parameter. Indeed, baseline and lowest rScO_2_ values were lower and maximal percentage decrease of rScO_2_ from baseline, AUT_50_ and AUT_80%base_ were greater in the low mean rScO_2_ group than in the high mean rScO_2_ group in this study (Supplementary Table S4). Moreover, patients in the low mean rScO_2_ group were accompanied by more frequent preoperative comorbidities than patients in the high mean rScO_2_ group. Therefore, a low mean rScO_2_ value seems to reflect the preoperative characteristics of patients and indicates increased risk of perioperative renal injury in OPCAB patients. Moreover, it is of particular importance that the mean rScO_2_ values remained low despite routine application of attempts to restore the values within 20% of the baseline and showed association with increased risk of AKI. This further strengthened the prognostic importance of intraoperative rScO_2_ monitoring identifying a high-risk group that would not respond to conventional means to increase oxygen delivery to the tissues, which would otherwise be not detected by other monitoring devices.

In this study, the cutoff low mean rScO_2_ value (58.5%) was close to the median baseline value of the low mean rScO_2_ group (57%). In line with these results, institutional protocols for restoring rScO_2_ reduction to less than 20% of baseline were universally applied. Still, low mean rScO_2_ was associated with increased AKI occurrence, possibly because the baseline rScO_2_ value was 57% and below the cutoff of 58.5%. In addition, although we cannot assure protocol adherence because of the retrospective nature of the study, patients in the low mean rScO_2_ group were treated with more frequent intraoperative vasopressors than in the high mean rScO_2_ group, indicating the efforts to increase rScO_2_. Notably, preoperative Hb concentrations were lower, the proportion of patients with anemia was higher, and intraoperative Hb concentrations were lower at all measurement points in the low mean rScO_2_ group than in the high mean rScO_2_ group (Supplementary Table S5). As our transfusion trigger was a Hb concentration less than 8 g/dL, it is difficult to deduce whether transfusion above this trigger based on rScO_2_ level resulted in an increase in mean rScO_2_ or improved the outcome, which are beyond the scope of this study.

The limitations of the present study are related to its retrospective nature. In addition, it is not known whether low mean rScO_2_ was the result of either nonadherence or nonresponse to the rScO_2_ management algorithm, although we tried to routinely apply the algorithm to restore the decrease of rScO_2_ according to the protocol in our clinical practice. In this regard, we only intended to observe the relationship between rScO_2_ and AKI. In a previous study, low rScO_2_ revealed prognostic value regardless of the response to the management algorithm, which suggested the usefulness of rScO_2_ for risk stratification [10]. In addition, as a limitation caused by the characteristics of the retrospective study, hemodynamic data such as systemic pressure, cardiac index, and SvO_2_ that could affect rScO_2_ were not continuously collected in the same way as rScO_2_, and the relationship between hemodynamic data and rScO_2_ could not be analyzed. Nonetheless, this study included as much pre- and intraoperative data as possible to analyze rScO_2_ values and postoperative AKI. Additionally, automatic and continuous collection of rScO_2_ values before induction of anesthesia to the end of surgery and inclusion of data from a relatively large number of patients compared to previous studies are strengths of the current study. Moreover, the current study provides primary evidence regarding the association of low intraoperative mean rScO_2_ and AKI specific to OPCAB, despite efforts to restore the rScO_2_ values within 20% of the baseline value, allowing identification of patients at risk of AKI, and possibly advocate early preventive measures of AKI in selected patients who would benefit the most.

## 5. Conclusions

In this retrospective analysis conducted in OPCAB patients, low intraoperative mean rScO_2_ value was independently associated with postoperative AKI. As it is a noninvasive form of monitoring with virtually no limitations in its application, early initiation of KDIGO bundles should be investigated in high-risk patients based on intraoperative mean rScO_2_ of AKI incidence and prognosis in OPCAB.

## Figures and Tables

**Table 1:** Preoperative demographic and morphometric data.

	Total (*n* =580)	Non-AKI (*n* = 437, 75.3%)	AKI (*n* = 143, 24.7%)	*p*-Value
Patient characteristics				
Age, years	67 (61–73)	67 (61–72)	69 (63–75)	0.004
Female, *n*	132 (22.8%)	93 (21.3%)	39 (27.3%)	0.138
Weight, kg	66.0 (59.8–73.0)	66.0 (60.0–72.8)	64.0 (57.3–73.5)	0.068
EuroSCORE II, %	1.18 (0.80–2.00)	1.10 (0.76–1.88)	1.50 (1.00–2.19)	<0.001
Comorbid medical disease				
Hypertension, *n*	409 (70.5%)	300 (68.7%)	109 (76.2%)	0.085
Diabetes mellitus, *n*	291 (50.2%)	213 (48.7%)	78 (54.6%)	0.228
Chronic kidney disease, *n*	55 (9.5%)	26 (6.0%)	29 (20.3%)	<0.001
MI within 1 week, *n*	69 (11.9%)	42 (9.6%)	27 (18.9%)	0.003
MI within 1 month, *n*	114 (19.7%)	77 (17.6%)	37 (25.9%)	0.031
MI within 3 months, *n*	120 (20.7%)	83 (19.0%)	37 (25.9%)	0.078
Unstable angina, *n*	189 (32.6%)	143 (32.7%)	46 (32.2%)	0.902
Congestive HF, *n*	97/575 (16.9%)	70/433 (16.2%)	27/142 (19.0%)	0.432
LVEF < 40%, *n*	79/574 (13.8%)	51/431 (11.8%)	28 (19.6%)	0.020
Left main disease, *n*	90 (15.5%)	72 (16.5%)	18 (12.6%)	0.265
Cerebrovascular accident, *n*	86 (14.8%)	57 (13.0%)	29 (20.3%)	0.035
PAOD, *n*	29 (5.0%)	17 (3.9%)	12 (8.4%)	0.032
Liver cirrhosis, *n*	5 (0.9%)	3 (0.7%)	2 (1.4%)	0.601
Laboratory related				
Creatinine, mg/dL	0.88 (0.75–1.05)	0.87 (0.75–1.03)	0.92 (0.75–1.14)	0.101
Anemia, *n*	255 (44.0%)	175 (40.1%)	80 (55.9%)	<0.001
Alb, g/dL	4.2 (3.8–4.4)	4.2 (3.9–4.5)	4.0 (3.7–4.2)	<0.001
CRP, mg/L	1.5 (0.6–4.5)	1.4 (0.5–4.2)	1.6 (0.8–6.2)	0.052
Medication				
RAS, *n*	320 (55.2%)	241 (55.2%)	79 (55.2%)	0.984
Beta blocker, *n*	318 (54.8%)	237 (54.2%)	81 (56.6%)	0.615
Calcium channel blocker, *n*	239 (41.3%)	171 (39.2%)	68 (47.6%)	0.079
Statin, *n*	490 (84.5%)	374 (85.6%)	116 (81.1%)	0.201
Emergency surgery, *n*	28 (4.8%)	12 (2.8%)	16 (11.2%)	<0.001
Intraoperative clinical data				
Duration of surgery, min	233 (210–252)	233 (210–255)	233 (215–248)	0.885
Inotropic agent requirement, *n*	76 (13.1%)	49 (11.2%)	27 (18.9%)	0.018
Vasopressin requirement, *n*	323 (55.7%)	231 (52.9%)	92 (64.3%)	0.017
Transfusion (pRBC), *n*	98 (16.9%)	55 (12.6%)	43 (30.1%)	<0.001
Cell saver volume, mL	220 (210–246)	220 (210–240)	223 (212–250)	0.178
Urine output, mL	250 (150–380)	250 (150–400)	200 (90–360)	0.001
Fluid intake, 100 mL	19.0 (15.0–22.5)	19.0 (15.0–22.5)	19.0 (15.0–23.0)	0.820
Cardiac index < 2.0 L/min/m^2^, *n*	499 (86.0%)	371 (84.9%)	128 (89.5%)	0.167
SvO_2_ < 65%, *n*	145 (25.0%)	100 (22.9%)	45 (31.5%)	0.040

Values are presented as median (interquartile range), or number of patients (%). The denominator is shown when the sample sizes are different due to missing data. If no denominator is provided, all data were present. *p*-values obtained when comparing the AKI group to the non-AKI group. AKI: acute kidney injury; EuroSCORE II: European System for Cardiac Operative Risk Evaluation II; MI: myocardial infarction; congestive HF: congestive heart failure defined by NYHA III or IV, NYHA, New York Heart Association Functional Classification; LVEF < 40%: left ventricular ejection fraction under 40%; left main disease: left main coronary artery disease; PAOD: peripheral arterial occlusive disease; CRP: C-reactive protein; RAS: renin–angiotensin-system (RAS)-acting agents; pRBC: packed erythrocytes; SvO_2_: mixed venous oxygen saturation.

**Table 2:** Intraoperative cerebral oximetry parameters.

	Non-AKI (n = 437, 75.3%)	AKI (n = 143, 24.7%)	*p*-Value	AUC
rScO_2_ data				
Baseline rScO_2_, %	61 ± 8	58 ± 9	<0.001	0.593
Mean rScO_2_, %	59 (54–64)	55 (50–61)	<0.001	0.636
Lowest rScO_2_, %	46 (40–52)	42 (36–49)	<0.001	0.618
Maximal percent decrease of rScO_2_ from baseline, %	24 (18–32)	27 (19–36)	0.029	0.561
AUT_50_, 10 min%	0.7 (0–26.8)	14.7 (0.1–71.4)	<0.001	0.620
AUT_80%base_, 10 min%	1.3 (0–22.4)	2.7 (0.1–31.1)	0.116	0.543
rScO_2_ < 50%, n	281 (64.3%)	111 (77.6%)	0.003	0.567
rScO_2_ < 80%base, n	286 (65.5%)	106 (74.1%)	0.054	0.543

Values are presented as mean ± SD, median (interquartile range), or number of patients (%). *p*-values obtained when comparing the AKI group to the non-AKI group. AKI: acute kidney injury; AUC: area under the ROC curve; rScO_2_: cerebral regional oxygen saturation; AUT_50_: area under the threshold below an absolute value of 50% of rScO_2_; AUT_80%base_: area under the threshold below 80% of baseline rScO_2_; 80%base: 80% of baseline rScO_2_ value.

**Table 3:** Multivariate logistic regression analysis for acute kidney injury.

Variables *	Adjusted OR	95% CI	*p*-Value
Age, per 1-year increase	1.020	0.994–1.046	0.137
Chronic kidney disease, yes	3.126	1.697–5.758	<0.001
MI within 1 week, yes	1.828	0.984–3.397	0.057
Anemia, yes	0.828	0.513–1.338	0.442
Albumin, per 1 g/dL increase	0.645	0.394–1.056	0.081
Emergency surgery, yes	3.300	1.405–7.750	0.006
Transfusion (pRBC), yes	1.641	0.948–2.841	0.077
Mean rScO_2_, per 1% increase	0.964	0.937–0.990	0.008

* The known risk factors and variables with a *p*-value < 0.01 from univariate analysis (Supplementary Table S2) were included in this multivariate logistic regression analysis. OR: odds ratio; CI: confidence interval; MI: myocardial infarction; pRBC: packed erythrocytes; rScO_2_: regional cerebral oxygen saturation.

**Table 4:** Postoperative outcomes based on cutoff value for mean rScO_2_.

Postoperative Outcome	High Mean rScO_2_	Low Mean rScO_2_ *	*p*-Value
Acute kidney injury, *n*	45 (16.1%)	98 (32.6%)	<0.001
Stage of acute kidney injury **			<0.001
Stage 1, *n*	41(14.7%)	85 (28.2%)	
Stage 2, *n*	2 (0.7%)	5 (1.7%)	
Stage 3, *n*	2 (0.7%)	8 (2.7%)	
Cerebrovascular accident, *n*	0 (0.0%)	11 (3.7%)	0.001
Delirium, *n*	31 (11.1%)	68 (22.6%)	<0.001
Re-operation, *n*	4 (1.4%)	6 (2.0%)	0.754
Sternal infection, *n*	2 (0.7%)	6 (2.0%)	0.289
Mechanical ventilation > 24 h, *n*	10 (3.6%)	9 (3.0%)	0.688
Myocardial infarction, *n*	6 (2.2%)	12 (4.0%)	0.203
30-day or in-hospital mortality, *n*	1 (0.4%)	7 (2.3%)	0.070
Intensive care unit days, day	3 (3–3)	3 (3–4)	0.007
Hospital days after surgery, day	8 (7–10)	9 (8–12)	<0.001

Values are presented as the number of patients (%) or median (interquartile range). *p*-values obtained when comparing the high mean rScO_2_ group to the low mean rScO_2_ group. * Low mean rScO_2_ was defined as rScO_2_ < 58.5% and the optimal cutoff value for the mean rScO_2_ was determined by AUROC analysis and the Youden’s index. ** Acute kidney injury staging 1 to 3 according to the classification proposed by Kidney Disease: Improving Global Outcomes (KDIGO). rScO_2_: regional cerebral oxygen saturation.

## Data Availability

Data are available upon request to corresponding author.

## Supplementary Materials

The following supporting information can be downloaded at: https://www.mdpi.com/article/10.3390/jcm12010359/s1, Table S1: Postoperative outcomes based on the development of AKI; Table S2: Univariate logistic regression analysis for acute kidney injury; Table S3: Demographic and Morphometric Data based on cutoff value for mean rScO_2_; Table S4: rScO_2_ values in the high and low mean rScO_2_ group; Table S5: Variables in arterial blood gas analysis.
