# Supplementary material for: Low Intraoperative Cerebral Oxygen Saturation Is Associated with Acute Kidney Injury after Off-Pump Coronary Artery Bypass
Source: J Clin Med. 2023 Jan 2;12(1):359. doi: 10.3390/jcm12010359 (PMC9821185; doi:10.3390/jcm12010359)
Supplement: Supplementary file 1 [file jcm-12-00359-s001.zip › Supplementary Tables final revised.pdf]

Supplementary Table S1. Postoperative outcomes based on the development of AKI

Postoperative outcome	**Total (*n* =580)**	**Non-AKI (*n*=437)**	**AKI (*n*=143)**	***p*-Value**
Cerebrovascular accident, *n*	11 (1.9%)	7 (1.6%)	4 (2.8%)	0.478
Delirium, *n*	99 (17.1%)	58 (13.3%)	41 (28.7%)	< 0.001
Re-operation, *n*	10 (1.7%)	5 (1.1%)	5 (3.5%)	0.072
Sternal infection, *n*	8 (1.4%)	4 (0.9%)	4 (2.8%)	0.108
Mechanical ventilation > 24 h, *n*	19 (3.3%)	10 (2.3%)	9 (6.3%)	0.028
Myocardial infarction, *n*	18 (3.1%)	14 (3.2%)	4 (2.8%)	1.000
30-day or in-hospital mortality, *n*	8 (1.4%)	1 (0.2%)	7 (4.9%)	< 0.001
Intensive care unit days, days	3 (3-4)	3 (3-3)	3 (3-5)	< 0.001
Hospital days after surgery, days	9 (8-11)	8 (7-10)	11 (8-15)	< 0.001

Values are presented as the number of patients (%) or median (interquartile range).
p-values obtained when comparing the AKI group to the non-AKI group.
AKI, acute kidney injury.

Supplementary Table S2. Univariate logistic regression analysis for acute kidney injury

	Univariate analysis		
	**Crude OR**	**95% CI**	***p*-Value**
Preoperative clinical data			
Age, per 1 year increase	1.033	1.010-1.057	0.005
Female, yes	1.387	0.899-2.140	0.139
EuroSCORE II, per 1% increase	1.116	1.022-1.220	0.015
Hypertension, yes	1.464	0.948-2.261	0.086
Diabetes mellitus, yes	1.262	0.864-1.843	0.229
Chronic kidney disease, yes	4.021	2.277-7.100	< 0.001
MI within 1 week, yes	2.189	1.294-3.704	0.004
Congestive HF, yes	1.218	0.745-1.989	0.432
LVEF < 40%, yes	1.814	1.094-3.009	0.021
Cerebrovascular accident, yes	1.696	1.035-2.778	0.036
PAOD, yes	2.263	1.054-4.861	0.036
Anemia, yes	1.901	1.298-2.784	0.001
Albumin, per 1 g/dL increase	0.378	0.251-0.569	< 0.001
CRP, per 1 mg/L increase	1.010	0.997-1.024	0.136
Emergency surgery, yes	4.462	2.057-9.678	< 0.001
Intraoperative clinical data			
Inotropic agent requirement, yes	1.843	1.103-3.080	0.020
Vasopressin requirement, yes	1.609	1.089-2.377	0.017
Transfusion (pRBC), yes	2.987	1.894-4.711	< 0.001
Urine output, per 1 ml increase	0.999	0.998-1.000	0.022
Cardiac index < 2.0 L/min/m^2, yes	1.518	0.837-2.754	0.170
SvO_2 < 65%, yes	1.547	1.019-2.349	0.040
$rScO_2$ data			
Baseline $rScO_2$, per 1% increase	0.956	0.935-0.978	< 0.001
Mean $rScO_2$, per 1% increase	0.944	0.922-0.966	< 0.001
Lowest $rScO_2$, per 1% increase	0.959	0.940-0.978	< 0.001
Maximal percent decrease of $rScO_2$ from baseline, per 1% increase	1.015	1.000-1.030	0.057
AUT_{50}, per 10 min% increase	1.004	1.002-1.007	< 0.001
$rScO_2$ < 50%, yes	1.926	1.241-2.988	0.004
$rScO_2$ < 80%base, yes	1.513	0.991-2.309	0.055

OR, odds ratio; CI, confidence interval; EuroSCORE II, European System for Cardiac Operative Risk Evaluation II; MI, myocardial infarction; Congestive HF, congestive heart failure defined by NYHA III or IV; NYHA, New York Heart Association Functional Classification; LVEF < 40%, left ventricular ejection fraction under 40%; PAOD, peripheral arterial occlusive disease; CRP, C-reactive protein; pRBC, packed erythrocytes; SvO_2, mixed venous oxygen saturation; $rScO_2$, regional cerebral oxygen saturation; AUT_{50}, area under the threshold below an absolute value of 50% of $rScO_2$; 80%base, 80% of baseline $rScO_2$ value.

Supplementary Table S3. Demographic and Morphometric Data based on cut-off value for mean $rScO_2$

	**High mean $rScO_2$ (n = 279)**	**Low mean $rScO_2$* (n = 301)**	***p*-Value**
Preoperative clinical data			
Age, years	64 (59-71)	69 (63-75)	< 0.001
Female, *n*	38 (13.6%)	94 (31.2%)	< 0.001
EuroSCORE II, %	0.99 (0.71-1.50)	1.42 (0.92-2.25)	< 0.001
Hypertension, *n*	190 (68.1%)	219 (72.8%)	0.219
Diabetes mellitus, *n*	111 (39.8%)	180 (59.8%)	< 0.001
Chronic kidney disease, *n*	14 (5.0%)	41 (13.6%)	< 0.001
MI within 1 week, *n*	32 (11.5%)	37 (12.3%)	0.760
Congestive heart failure, *n*	31/278 (11.2%)	66/297 (22.2%)	< 0.001
LVEF < 40%, *n*	28/275 (10.2%)	51/299 (17.1%)	0.017
PAOD, *n*	12 (4.3%)	17 (5.7%)	0.457
Cerebrovascular accident, *n*	36 (12.9%)	50 (16.6%)	0.209
Liver cirrhosis, *n*	1 (0.4%)	4 (1.3%)	0.375
Anemia, *n*	75 (26.9%)	180 (59.8%)	< 0.001
Albumin, g/dL	4.3 (4.0-4.5)	4.0 (3.7-4.3)	< 0.001
Emergency, *n*	12 (4.3%)	16 (5.3%)	0.569
**Intraoperative clinical data**			
Duration of surgery, min	236 (211-256)	229 (210-249)	0.136
Inotropic agent requirement, *n*	29 (10.4%)	47 (15.6%)	0.063
Vasopressin requirement, *n*	140 (50.2%)	183 (60.8%)	0.010
Transfusion (pRBC), *n*	14 (5.0%)	84 (27.9%)	< 0.001
Hemoglobin concentration before transfusion, g/dL	8.3 (7.9-8.7)	8.5 (8.0-8.9)	0.506
Cell saver volume, ml	220 (210-240)	220 (210-250)	0.926
Urine output, ml	250 (150-380)	250 (130-380)	0.182
Fluid intake, 100 ml	19.0 (14.8-22.5)	18.5 (15.0-23.0)	0.600
Cardiac index < 2.0 L/min/m^2, *n*	230 (82.4%)	269 (89.4%)	0.016
SvO_2 < 65%, *n*	52 (18.6%)	93 (30.9%)	< 0.001

Values are presented as median (interquartile range), or number of patients (%).

The denominator is shown when the sample sizes are different due to missing data. If no de-nominator is given, all data were present.

p-values obtained when comparing the high mean $rScO_2$ group to the low mean $rScO_2$ group.

* Low mean $rScO_2$ was defined as $rScO_2$ < 58.5% and the optimal cut-off value for the mean $rScO_2$ was determined by AUROC analysis and the Youden's index.

$rScO_2$, regional cerebral oxygen saturation; EuroSCORE II, European System for Cardiac Operative Risk Evaluation II; MI, myocardial infarction; Congestive heart failure, defined by NYHA III or IV; NYHA, New York Heart Association Functional Classification; LVEF<40%, left ventricular ejection fraction under 40%; PAOD, peripheral arterial occlusive disease; pRBC, packed erythrocytes; SvO_2, mixed venous oxygen saturation.

Supplementary Table S4. $rScO_2$ values in the high and low mean $rScO_2$ group

$rScO_2$ data	**High mean $rScO_2$ (*n* = 279)**	**Low mean $rScO_2$*** **(*n* = 301)**	***p*-Value**
Baseline $rScO_2$, %	65 (60-70)	57 (52-62)	< 0.001
Lowest $rScO_2$, %	52 (46-56)	41 (35-45)	< 0.001
Maximal percent decrease of $rScO_2$ from baseline, %	21 (16-28)	29 (21-38)	< 0.001
AUT_{50}, 10 min%	0.0 (0.0-0.4)	24.7 (3.7-96.4)	< 0.001
$AUT_{80\%Base}$, 10 min%	0.6 (0.0-14.9)	5.8 (0.1-37.8)	< 0.001
rScO2 < 50%, *n*	105 (37.6%)	287 (95.3%)	< 0.001
rScO2 < 80%Base, *n*	155 (55.6%)	237 (78.7%)	< 0.001

Values are presented as median (interquartile range), or number of patients (%).
p-values obtained when comparing the high mean $rScO_2$ group to the low mean $rScO_2$ group.
* Low mean $rScO_2$ was defined as $rScO_2$ < 58.5% and the optimal cut-off value for the mean $rScO_2$ was determined by AUROC analysis and the Youden's index.
$rScO_2$, regional cerebral oxygen saturation; AUT_{50}, area under the threshold below an absolute value of 50% of $rScO_2$; $AUT_{80\%base}$, area under the threshold below 80% of baseline $rScO_2$, 80%base, 80% of baseline $rScO_2$ value.

Supplementary Table S5. Variables in arterial blood gas analysis

	**High mean $rScO_2$ (n = 279)**	**Low mean $rScO_2$* (n = 301)**	***p*-Value**
Post-induction			
Hb, g/dL	12.0 (11.4-12.8)	11.0 (10.2-11.6)	< 0.001
$PaCO_2$, mmHg	34.7 (32.7-36.8)	34.4 (32.3-36.6)	0.191
PaO_2, mmHg	180.1 (157.9-200.8)	183.8 (153.9-202.3)	0.428
During main grafting			
Hb, g/dL	11.0 (10.3-11.9)	9.7 (8.9-10.7)	< 0.001
$PaCO_2$, mmHg	36.8 (35.2-38.8)	36.3 (34.3-38.6)	0.183
PaO_2, mmHg	194.4 (176.3-210.5)	197.3 (177.7-212.5)	0.330
Sternal closure			
Hb, g/dL	10.6 (9.9-11.5)	9.6 (8.9-10.4)	< 0.001
$PaCO_2$, mmHg	36.8 (34.5-38.9)	36.8 (34.6-38.9)	0.994
PaO_2, mmHg	176.3 (144.4-199.8)	181.1 (152.5-204.0)	0.045
Immediate Postop.			
Hb, g/dL	10.7 (10.0-11.5)	9.8 (9.2-10.5)	< 0.001
$PaCO_2$, mmHg	34.7 (32.2-37.6)	34.7 (32.6-37.1)	0.719
PaO_2, mmHg	150.7 (126.4-175.3)	152.6 (130.5-180.3)	0.263

Values are presented as median (interquartile range).

p-values obtained when comparing the high mean $rScO_2$ group to the low mean $rScO_2$ group.

* Low mean $rScO_2$ was defined as $rScO_2$ < 58.5% and the optimal cut-off value for the mean $rScO_2$ was determined by AUROC analysis and the Youden's index.

Hb, hemoglobin; $PaCO_2$, arterial partial pressure of CO_2; PaO_2, arterial partial pressure of O_2; Immediate Postop., immediate postoperative phase.
